# Serious Game for Change in Behavioral Intention Toward Lifestyle-Related Diseases: Experimental Study With Structural Equation Modeling Using the Theory of Planned Behavior

**DOI:** 10.2196/28982

**Published:** 2022-02-21

**Authors:** Mahiro Egashira, Daisuke Son, Arisa Ema

**Affiliations:** 1 Division of Science Interpreter Training Program Komaba Organization for Educational Excellence The University of Tokyo Tokyo Japan; 2 Department of Community-Based Family Medicine Faculty of Medicine Tottori University Yonago Japan; 3 Institute for Future Initiatives The University of Tokyo Tokyo Japan

**Keywords:** lifestyle-related disease, mechanism of behavior change, serious game, theory of planned behavior

## Abstract

**Background:**

Health activities should be tailored to individual lifestyles and values. To raise awareness of health behaviors, various practices related to health education, such as interactive activities among individuals with different backgrounds, have been developed. Moreover, serious games have been used as a tool for facilitating communication. However, there have been few investigations that are based on the framework of the theory of planned behavior on the mechanisms of health-related behavioral intention change from playing serious games.

**Objective:**

We aimed to investigate the mechanisms of behavioral intention change among various age groups after an intervention using a serious game to increase awareness of lifestyle-related diseases.

**Methods:**

Adults, undergraduates, and high school students played a serious game, called Negotiation Battle, and answered a questionnaire—Gaming Event Assessment Form for Lifestyle-related Diseases—before, immediately after, and 2-4 weeks after the game. The questionnaire was composed of 16 items based on the theory of planned behavior. We used structural equation modeling to compare responses from the 3 groups.

**Results:**

For all 3 age groups (adults: mean 43.4 years, range 23-67 years; undergraduates: mean 20.9 years, range 19-34 years; high school students: mean 17.9 years, 17-18 years), perceived behavior control was the key factor of behavioral intention change. Immediately after the game, causal relationships between perceived behavioral control and behavioral intention were enhanced or maintained for all groups—adults (before: path coefficient 1.030, *P*<.001; after: path coefficient 2.045, *P*=.01), undergraduates (before: path coefficient 0.568, *P*=.004; after: path coefficient 0.737, *P*=.001), and high school students (before: path coefficient 14.543, *P*=.97; after: path coefficient 0.791, *P*<.001). Analysis of free descriptions after intervention suggested that experiencing dilemma is related to learning and behavioral intention.

**Conclusions:**

The study revealed that the serious game changed the behavioral intention of adolescents and adults regarding lifestyle-related diseases, and changes in perceived behavioral control mediated the alteration mechanism.

## Introduction

Noncommunicable diseases, such as cardiovascular disease, cancer, diabetes, and chronic respiratory disease, are the leading cause of mortality worldwide and accounted for 71% of 41 million deaths in 2018 [1]. The major risk factors of mortality that contribute to noncommunicable diseases and are modifiable, given effective interventions [2], are high blood pressure, tobacco use, high blood glucose levels, physical inactivity, and being overweight or obese. In Japan, the current leading causes of death are malignant neoplasm, heart disease, and cerebrovascular diseases, which accounted for more than 50% of total deaths in 2017 [3]. Therefore, preventing deaths due to lifestyle-related diseases is a major concern. In Japan, lifestyle-related diseases cause major medical and economic problems [4]. Notably, however, lifestyle-related diseases are primarily dependent on individual values and attitudes and it is difficult to intervene. To prevent such diseases, people must balance unhealthy and healthy behaviors while conforming to their values and lifestyle. To encourage awareness and behavior changes, methods of health communication, such as interactive dialog between individuals with different backgrounds, have been proposed [5-7]. Applying a combination of health communication models, including serious games, has proven effective in improving knowledge and self-management [8,9].

Serious games are useful as a communication tool. They are designed for teaching, training, and changing knowledge, attitudes, and behavior while remaining entertaining [10]. Moreover, the design and practicability of serious games have been evaluated in the fields of health care [8-10]. Although serious games are simulated, they can provide real-world experiences to participants through role-playing [11].

The theory of planned behavior is a hypothesis on health behavior [12-14] and has been widely applied to motivation analyses of health-related behaviors [15-17]. The theory of planned behavior postulates that 3 factors influence behavioral intention: (1) attitude toward behavior, that is, the belief that healthy behavior leads to health and appreciation of the consequences of such behavior; (2) the subjective norm, the realization that other people believe that healthy behavior is desirable and conform to this social expectation; and (3) perceived behavioral control, which is the belief that one possesses the resources and skills necessary for healthy behavior. Therefore, the theory of planned behavior postulates that individuals are likely to engage in a health behavior if they believe that (1) it will lead to particular outcomes they appreciate, (2) people important to them think they should engage, and (3) they have the necessary resources and opportunities to perform the behavior.

Many studies [18-20] have reported on the development of serious games based on theory of planned behavior that target chronic diseases or disease prevention and have mainly focusing on the game design and the interventions. Serious games have been designed on the basis of behavioral models to highlight chronic diseases in children [18]; for the prevention and rehabilitation of diseases, such as asthma, diabetes, or HIV [19]; or to encourage healthy lifestyles [20]. However, to date, no studies have investigated the mechanism of behavioral intention change through serious games using the theory of planned behavior framework as a basis. Moreover, little is known about differences in the effects of health-targeted serious games on various age groups. To address this research gap, we aimed to identify on which behavior change mechanisms a serious game for lifestyle-related diseases has an effect and to elucidate differences according to various age groups.

Serious games are frequently used in combination with educational activities, especially in health care fields, with positive outcomes [21,22]. For example, serious games targeting health behaviors can improve cognitive abilities in older adults [23] or improve neuropsychological abilities of alcoholic patients [24]. Unlike regular computer and video games, serious games have dual goals of entertaining and promoting behavior change [10]. Face-to-face serious games (eg, board games) combined with health education may also achieve similar benefits [25].

## Methods

### Negotiation Battle

**Figure 1:**
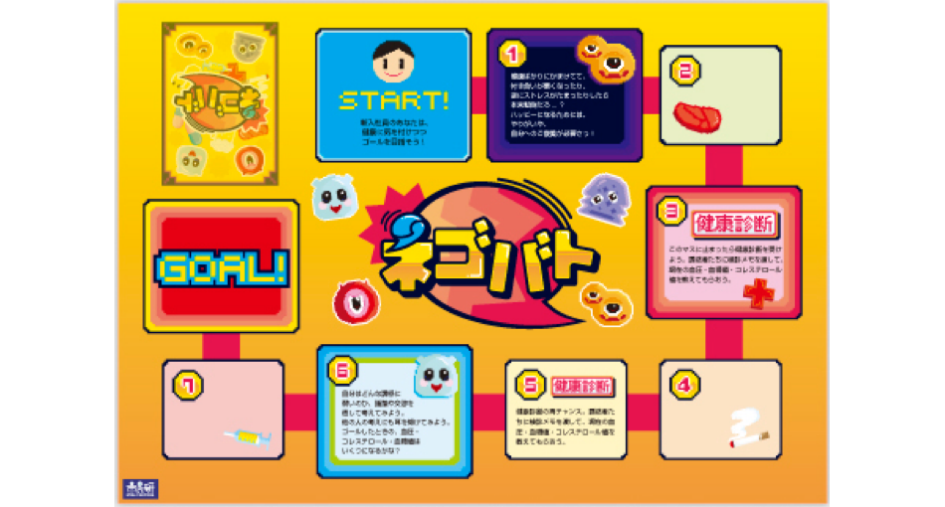
Negotiation Battle serious game.

We employed a serious game called *Negotiation Battle* (Figure 1), which is a board game developed by a nonprofit organization called Citizen’s Science Initiative Japan [26]. We chose Negotiation Battle because it is a board game in which 2 teams with different views on lifestyle-related diseases can discuss the issues while playing the game, and we thought it would have an educational effect through discussion.

**Figure 2:**
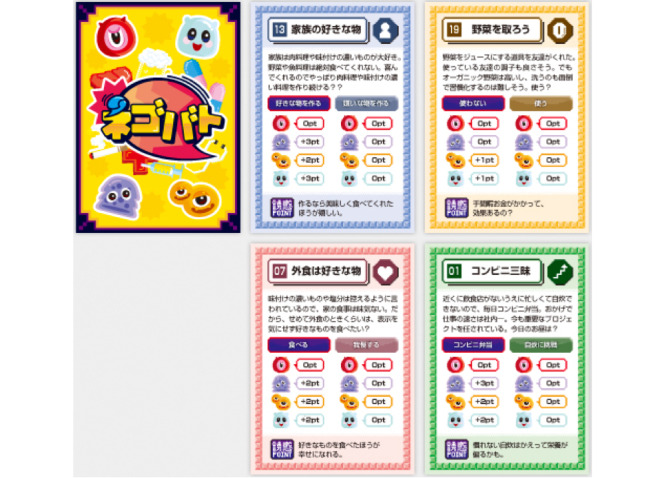
Dilemma cards regarding behaviors such as whether to season meals at home more, use a juicer to eat more vegetables, eat out at high-calorie restaurants, or eat convenience store lunches every day. Example: “07. I want to eat what I like when eating out.” You are told to avoid overly seasoned and salty foods, so your meals at home are tasteless. So, at least when you eat out, you want to eat what you want without worrying about the label. Do you eat like that? A: Yes, B: No. Temptation Tip: You'll be happier if you eat what you like.

**Figure 3:**
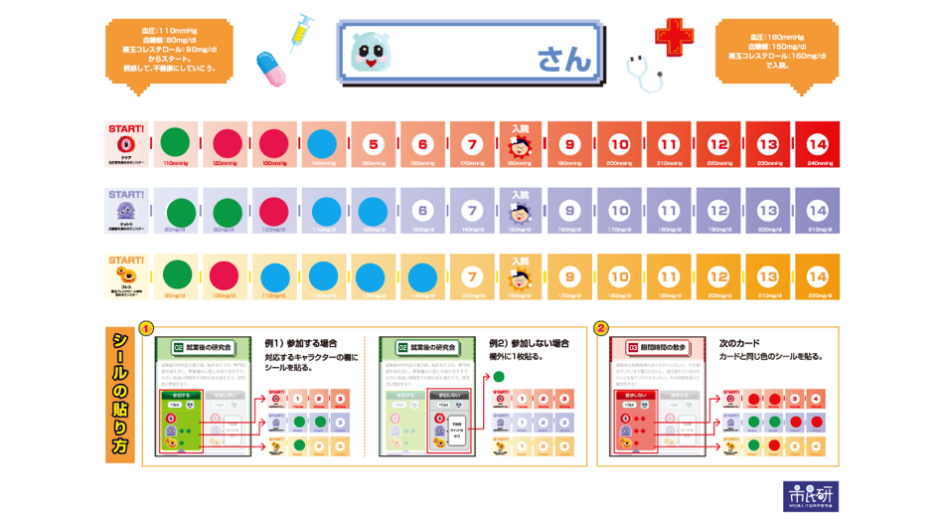
Health sheet with blood pressure, blood sugar, and cholesterol. Points accumulate depending on the results of each behavior. When points reach a threshold, the human is hospitalized, which is game over.

Negotiation Battle is played by 6 people on 2 sides, with one side playing the seducer (3 people) and one side playing the human (3 people). The seducer team persuades the human team, but the seducer team also exchange opinions with each other and with the human team. The duration of the game is 20 to 30 minutes per set, and the game set includes a dilemma card (Figure 2) and health sheet (Figure 3). For the dilemma situation, unhealthy points and happy points are listed on the card about each specific behavior. On the health sheet (one for each seducer role and only the seducer team can record on and view the sheet), unhealthy values of the human team are recorded. When the health points reach a certain value, the seducer is hospitalized.

The goal of the seducer is to add up the unhealthy points of the person he is in charge of, while the goal of the human is to accumulate happy points without being hospitalized. (1) The seducer reads out the card’s dilemma situation and seduces the human to focus on work, hobbies, and relationships and continue with behaviors that are unhealthy. (2) The human decides whether or not to accept the temptation while negotiating and interacting with the seducer and other human players. (3) If the human accepts the temptation, the seducer adds the value of the unhealthy points on the dilemma card to the health sheet at hand. (4) The human earns happy points. (5) After each human's decision is made on one card, the player moves on to the next card and repeats steps 1 through 4. The health sheet shows the threshold of ill health (ie, hospitalization, which is game over for the human), but the human cannot know the current value of ill-health points. (6) At the end of the game, the seducer discloses the information on the health sheet to the human, and (7) together they reflect on and discuss the types of temptations to which they were vulnerable. Thus, participants are stimulated to engage in interactive communication by applying the imaginary dilemma to real-life situations.

### Participants

We recruited participants from 3 age groups—adults (including postgraduate students), undergraduate students, and high school students—because behaviors and attitudes toward lifestyle-related diseases could differ between adults and young people. In addition, among young people, we expected that there would be differences between high school students, who mainly live with their parents, and university students, who are often independent from their parents (living alone).

Adults, who were invited to participate and applied through a social networking service (Facebook, mailing list), attended a total of 3 Negotiation Battle sessions held between November and December 2016. For university students, we invited second-year students of the Tokyo University to participate in January 2017, and for high school students, we invited third-year students of a high school in Tokyo to participate as part of their classes in January 2017. 

### Data Collection

The participants were asked to submit a questionnaire (Gaming Event Assessment Form for Lifestyle-related Diseases) before, immediately after, and 2 to 4 weeks after the intervention. Prior to the intervention, an instruction document that described the survey was given to each participant, with the questionnaire and a return envelope to be filled in 2 to 4 weeks after the intervention and returned by mail.

There is a rule of thumb regarding sample size in structural equation modeling analysis that the sample size should be at least 5 times the number of parameters [27]. In this study, the questionnaire had 16 items (on the theory of planned behavior), and the minimum required sample size was estimated to be 80. However, since this study was conducted in the context of actual classes for university students and high school students, feasibility was given priority, and it was considered inevitable that there would be some groups below that size in each age group.

### Questionnaire Composition

**Figure 4:**
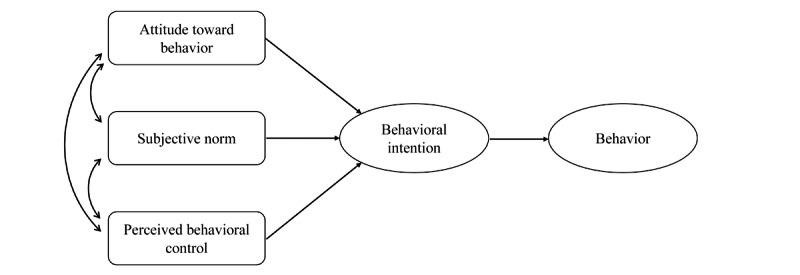
Theory of planned behavior diagram.

The questionnaire was constructed to assess components of the theory of planned behavior (Figure 4) to allow structural equation modeling of before-and-after comparisons, and additional items were inserted to obtain background information on the participants (age, family composition, occupation, and history of lifestyle-related diseases as a background profile of the participants). The questionnaire was scaled according to previous studies that examined 4 aspects, namely, attitude toward behavior [28], subjective norm [29], perceived behavioral control [30], and behavioral intention [31]. The questionnaire included additional items for free description of what the participants learned or whether they newly started healthy behaviors afterward: “What was your learning or awareness about healthy lifestyle?” (for both time points) and “What kind of healthy behaviors have you recently started?” (2 to 4 weeks after).

### Data Analysis

Responses were analyzed using SPSS Statistics and Amos software (version 23; IBM Corp). Structural equation modeling was performed to determine the relationship (causality or correlation) between factors of the theory of planned behavior. We used the comparative fit index (CFI), Tucker-Lewis index (TLI), and root mean square error of approximation (RMSEA) as fitness indices. Missing values were imputed (using means within each item). Statistical significance was set to *P*<.05. Content analysis was used for the free descriptions; characteristic concepts were extracted from entire free descriptions, and the frequency with which these concepts were observed was counted for each category.

### Ethical Consideration

Participants were given a written and verbal explanation of the study protocol, and only those who consented were included in the study. Anonymity was ensured; the contents of the questionnaire were viewed only by the researchers, and no identifiable information was disclosed. The researchers securely stored collected data. The undergraduate and high school students were assured that their responses would not place them at any academic disadvantage. Ethical procedures [32] of the Ministry of Education, Culture, Sports, Science, and Technology and Ministry of Health, Labor, and Welfare in Japan were followed; formal ethical approval is not mandated for this type of study under these guidelines.

## Results

### Participant Characteristics

The adult group ranged in age from 23 to 67 years, university students ranged in age from 19 to 34 years, and high school students ranged in age from 17 to 18 years (Table 1).

**Table 1:** Demographic characteristics.

Characteristic					Adults (n=22)			Undergraduate students (n=76)			High school students (n=24)
Age, mean (SD)					43.4 (14.4)			20.9 (2.6)			17.9 (0.3)
**Household, n (%)**											
	Living alone			4 (18)			38 (50)			0 (0)	
	Only a couple			3 (14)			1 (1)			0 (0)	
	Parent and child			11 (50)			30 (40)			22 (92)	
	Three generations			3 (14)			4 (5)			2 (8)	
	Unknown			1 (4)			3 (4)			0 (0)	
**Occupation, n (%)**											
	Medical and welfare specialists			7 (32)			—^a^			—	
	Nonmedical and welfare specialists			4 (18)			—			—	
	Graduate students			5 (23)			—			—	
	Others			5 (23)			—			—	
	Unknown			1 (4)			—			—	
**Experience of illness, n (%)**											
	**Self**										
		Positive	4 (18)			1 (1)			0 (0)		
		Negative	17 (77)			72 (95)			22 (92)		
		Unknown	1 (4)			3 (4)			2 (8)		
	**Family member**										
		Positive	8 (36)			22 (29)			1 (4)		
		Negative	12 (55)			50 (66)			23 (96)		
		Unknown	2 (9)			4 (5)			0 (0)		

^a^No data.

### Structural Equation Models of Behavioral Decision-making Mechanisms

**Figure 5:**
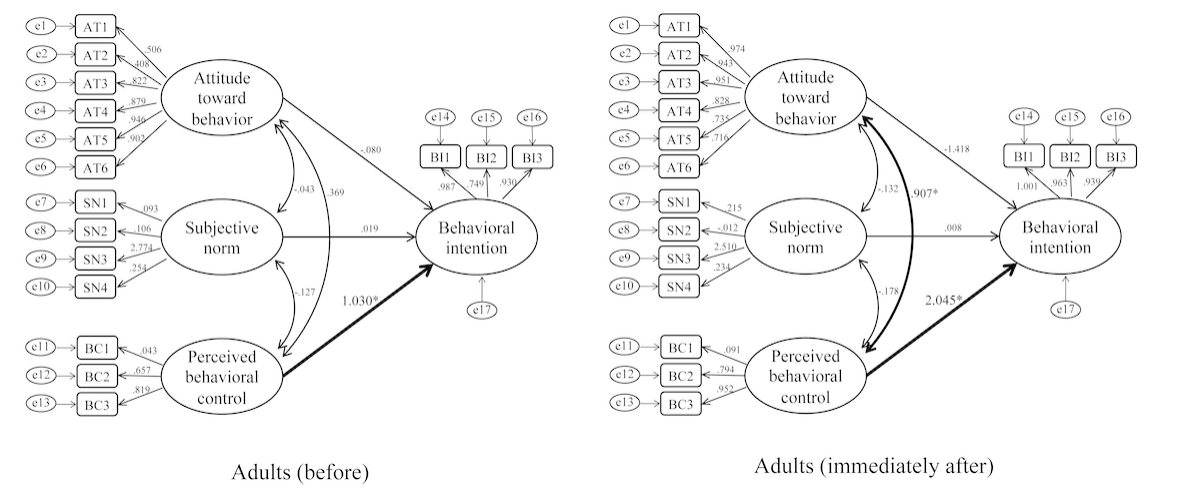
Structural equation model: adult participants before and immediately after participation. Standardized coefficients are shown on each path.

In adult participants, structural equation models demonstrated that there was a significant causal relationship between perceived behavioral control and behavioral intention both before (path coefficient 1.030, *P*<.001; CFI 0.562; TLI 0.464; RMSEA 0.259) and immediately after (path coefficient 2.045, *P*=.01; CFI 0.755, TLI 0.700, RMSEA 0.223) the intervention (Figure 5).

**Figure 6:**
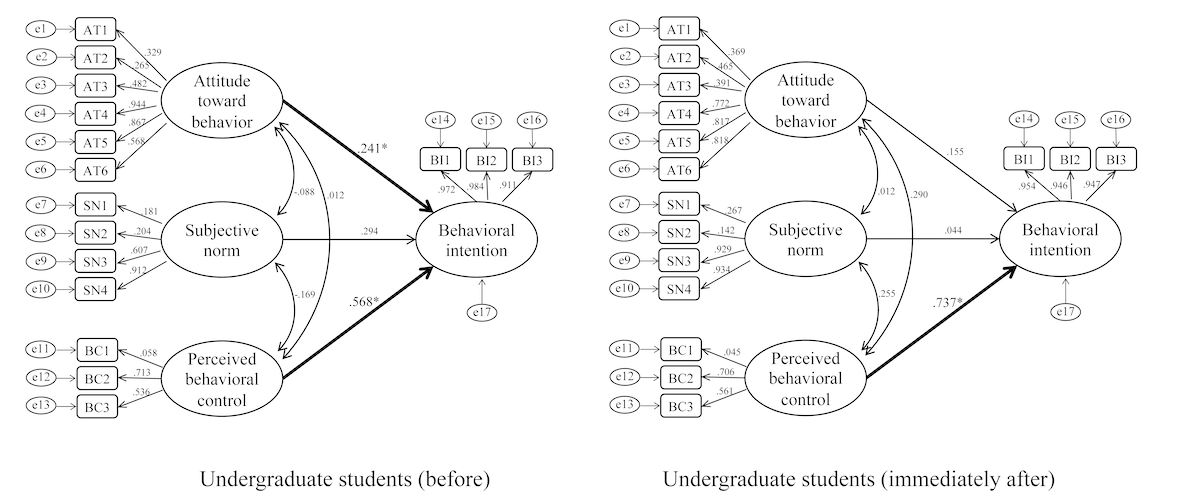
Structural equation model: undergraduate students before and immediately after participation. Standardized coefficients are shown on each path.

In undergraduate students, before the intervention, significant causal relationships between perceived behavioral control and behavioral intention (*P*=.004; CFI 0.781; TLI 0.731; RMSEA 0.138) and between attitude toward behavior and behavioral intention (*P*=.04) were evident. In contrast, however, the relationship between attitude toward behavior and behavioral intention was no longer significant immediately after the intervention (*P*=.22; CFI 0.785; TLI 0.701; RMSEA 0.140), which suggests that perceived behavioral control alone influences behavioral intention (Figure 6).

**Figure 7:**
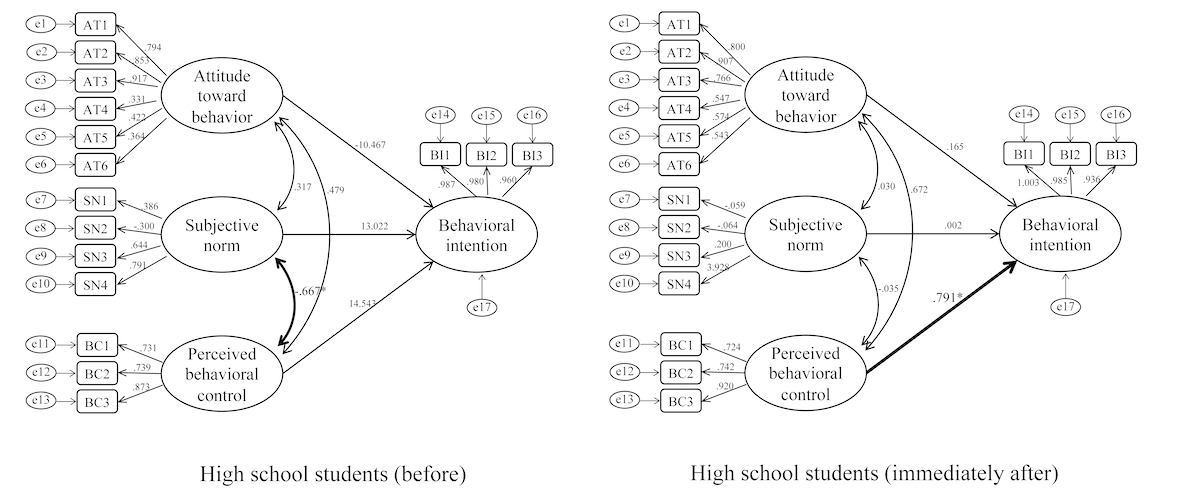
Structural equation model: high school students before and immediately after participation. Standardized coefficients are shown on each path.

In high school students, prior to the intervention, no factors significantly influenced behavioral intention (CFI 0.785, *P*=.97; TLI 0.701, *P*=.97; RMSEA 0.154, *P*=.97); however, a significant causal relationship (*P*<.001; CFI 0.709; TLI 0.596; RMSEA 0.210) was observed between perceived behavioral control and behavioral intention immediately after the intervention (Figure 7).

### Analysis of Free Descriptions

The number of valid responses for free descriptions immediately after the intervention were 20, 66, and 19 for adults, undergraduate students, and high school students, respectively; the number of valid responses for free descriptions 2 to 4 weeks after the interventions were 12, 54, and 16 for adults, undergraduate students, and high school students, respectively. A total of 8 concepts were observed (Table 2): dilemma, intention, learning, and status quo explanation with dilemma and learning description as dominant descriptions.

Responses 2 to 4 weeks after the intervention contained descriptions of behavior (adults: 10/17 concepts, 59%; undergraduates: 42/74 concepts, 57%; high school students: 14/18 concepts, 78%) with less descriptions of dilemma (adults: 3/17 concepts, 18%; undergraduates: 3/74 concepts, 4%; high school students: 2/18 concepts, 11%) or other concepts (Table 3).

**Table 2:** Extracted concepts and examples from free descriptions.

Concept	Sentence pattern	Examples
Dilemma	Want to do something but cannot	“When I prioritize fun activities and socializing, it leads to an action that is unhealthy in many cases.”
Intention	Want to continue doing something	“I want to start engaging in healthy activities and want to keep improving health awareness so that I can encourage others.”
Learning	Noticed something	“I came to realize my tendency to be worried about whether to prioritize career or health.”
Status quo explanation	Understood why	“I became aware that I am unhealthy.”
Behavior	Did something	“I started recording weights and diet using the body support app.”
Cognitive change	Adopted a new thought	“I became more positive about outlooks.”
Social pressure	Affected by others	“My health conditions could be affected by others.”
Game-related description	N/A^a^	“I want to use it in my study group.” “This game realizes us that health is a tradeoff with comfort rather than happiness.”

^a^N/A: not applicable.

**Table 3:** Concepts observed immediately after and 2 to 4 weeks after the game.

Concept	In Adults’ responses, n (%)		In undergraduate students’ responses, n (%)		In high school students’ responses, n (%)	
	Immediately after (n=44)	2 to 4 weeks after (n=17)	Immediately after (n=111)	2 to 4 weeks after (n=74)	Immediately after (n=30)	2 to 4 weeks after (n=18)
Dilemma	13 (30)	3 (18)	39 (35)	3 (4)	10 (33)	2 (11)
Intention	5 (11)	0 (0)	13 (12)	6 (8)	2 (7)	0 (0)
Learning	14 (32)	1 (6)	39 (35)	11 (15)	14 (47)	0 (0)
Status quo explanation	7 (16)	0 (0)	11 (10)	5 (7)	2 (7)	0 (0)
Behavior	0 (0)	10 (59)	0 (0)	42 (57)	0 (0)	14 (78)
Cognitive change	0 (0)	1 (6)	3 (3)	3 (4)	0 (0)	2 (11)
Social pressure	0 (0)	2 (12)	1 (1)	4 (5)	1 (3)	0 (0)
Game-related description	5 (11)	0 (0)	5 (5)	0 (0)	1 (3)	0 (0)

## Discussion

### Principal Findings

Our findings suggest that perceived behavioral control is a determinant of behavioral intention in adult participants who played Negotiation Battle, a game in which players face an imagined situation that induces dilemma and engage in dialog. Free descriptions revealed that adults frequently experienced dilemma (13/44, 30%) and learning (14/44, 32%), which were the expected characteristics of the game. Thus, it seems that exposure to different perspectives during dilemmas in the simulated scenarios and the subsequent dialog led to self-reflection and transformative learning, which reinforced their perceptions that they can control health-related behaviors.

Transformative learning pertains to “a deep, structural shift in basic premises of thought, feelings, and actions [33].” According to transformative learning theory, critical self-reflection on the assumptions of learners facing disorienting dilemma may occur, which leads them to explore new options regarding roles, relationships, and actions. After undergoing such phases, they build competence and self-confidence in new roles and relationships [34]. In the game, players faced imaginary dilemma situations, which may lead to critical self-reflection and cognitive change in their perceptions of their health behaviors.

For undergraduate students, 2 factors—namely, attitude toward behavior (path coefficient 0.241; *P*=.04) and perceived behavioral control (path coefficient 0.568; *P*=.004)—influenced behavioral intention prior to the intervention. Immediately after the intervention, the influence of perceived behavioral control on behavioral intention was maintained (path coefficient 0.737; *P*=.001), whereas that of attitude toward behavior was not (path coefficient 0.155; *P*=.22). This finding indicates that undergraduate students also reinforced perceived behavioral control toward healthy behavior by facing dilemmas and undergoing transformative learning.

For high school students, no significant factors for behavioral intention were observed prior to the intervention (*P*=.97). Afterward, perceived behavioral control contributed to behavioral intention (*P*<.001). Descriptions of dilemma (10/30, 33%) and learning (14/30, 47%) were mainly observed immediately after the intervention, whereas those of behavior (14/18, 78%) and cognitive change (2/18, 11.1%) appeared after 2 to 4 weeks, suggesting that playing Negotiation Battle triggered transformative learning in high school students as it did in adults and undergraduate students.

Notably, the serious game related to lifestyle-related diseases resulted in transformative learning even in high school students, with the majority of concepts (14/18, 78%) suggesting that participants started a new behavior after 2 to 4 weeks. All high school students who participated in the study lived with and were dependent on their parents; thus, we assumed that they would pay less attention to the context of their diet or health behaviors. However, our findings revealed that the game can increase health awareness even among high school students. This finding is in line with those of previous studies [35-37], which demonstrated that serious games designed for health behavior change can be effective for adolescents.

Moreover, we observed that Negotiation Battle reinforced perceived behavior control out of all factors of theory of planned behavior, which led to behavioral intention change. The findings of previous studies [38,39], that digital serious games for health promotion among older adults enhanced perceived behavioral control, support this.

### Strengths and Limitations

The study has 3 major strengths. First, we used the theory of planned behavior framework to reveal which factors led to change of behavioral intention after playing Negotiation Battle, which revealed that perceived behavioral control was a major influencing factor. Second, we found that Negotiation Battle can induce critical reflection and transformative learning by placing learners in simulated dilemmas. If transformative learning can be triggered, then the health consciousness transformed by learning will be likely sustained. Third, we compared the effects of Negotiation Battle on adults and on younger people (high school students); thus, the findings are observable across ages.

The study’s limitations are the relatively small sample size, which limits the generalizability of this study, and the lack of a control group in the study’s design to see the true effect of the intervention.

### Conclusions

Through the simulation of dilemma and dialog in Negotiation Battle, participants were encouraged to reflect on their health behaviors, and enhanced perceived behavioral control contributed to the change in health consciousness. Serious game interventions based on the framework of cognitive change processes appear to foster self-reflection and dialog, which encourages transformative learning and the improvement of specific lifestyle behaviors.

## Abbreviations

CFI: comparative fit index
HIV: human immunodeficiency virus
RMSEA: root mean square error of approximation
TLI: Tucker-Lewis index
